# Can Aluminum Affect Social Behavior and Cortisol Plasma Profile in the Neotropical Freshwater Teleost *Astyanax lacustris* (Teleostei: Characidae)?

**DOI:** 10.3390/life14121697

**Published:** 2024-12-21

**Authors:** Natália Pires Vieira Morais de Faria, Bruno Cavalheiro Araújo, Bianca Mayumi Silva Kida, Raisa Pereira Abdalla, Diego dos Santos Brito, Renata Guimarães Moreira, Renato Massaaki Honji

**Affiliations:** 1 Laboratório de Metabolismo e Reprodução de Organismos Aquáticos (LAMEROA), Departamento de Fisiologia, Instituto de Biociências, Universidade de São Paulo (IB/USP), Rua do Matão, trav. 14, No. 321, Cidade Universitária, São Paulo 05508-090, SP, Brazil; 2 Laboratório de Fisiologia e Nutrição de Organismos Aquáticos (LAFINUTRI), Núcleo Integrado de Biotecnologia, Universidade de Mogi das Cruzes, Avenida Dr. Cândido Xavier de Almeida e Souza, No. 200, Mogi das Cruzes 08701-970, SP, Brazil; 3 Laboratório de Aquicultura e Ecofisiologia Marinha (LAQUEFIM), Departamento de Fisiologia, Instituto de Biociências, Universidade de São Paulo (IB/USP), Rua do Matão, trav. 14, No. 321, Cidade Universitária, São Paulo 05508-090, SP, Brazil

**Keywords:** metal, endocrine disruption, behavioral disruption, glucocorticoids, xenobiotic

## Abstract

Aluminum (Al) can cause endocrine disruption in aquatic animals, but assessments of animal social behavior in neotropical teleost fish species with importance for Brazilian aquaculture have still not been addressed so far, which can further complete this ecotoxicological knowledge. In order to evaluate the social behavior and plasma cortisol concentration of fish exposed to Al, we performed a 1 h acute exposure with *Astyanax lacustris* couples in three different experimental groups: control in neutral pH (CTL/n group), acid pH (pH/ac group), and aluminum in acid pH (Al/ac group; 2.0 mg L^−1^). An ethogram of social interactions between males and females and swimming activities were performed. Furthermore, the cortisol plasma concentration was measured by enzyme-linked immunosorbent, and the gonadal maturation stage of the animals was evaluated by histology. Adult and mature females in the CTL/n and pH/ac groups were more aggressive and active than mature males, including several attacks on the male. Moreover, males did not present attack behavior in these groups at any time, but did show submission behavior and constant avoidance of female attacks. In the Al/ac, females did not attack males, couple decreased swimming activity, a repetitive movement toward the aquarium surface, and high mucus production were observed, making the water cloudy. Regarding cortisol plasma concentration, males had higher cortisol plasma concentrations than females in the CTL/n and pH/ac groups, which was not observed in the Al/ac group. Therefore, Al in addition to being described in the literature as an endocrine disruptor, it can be considered as behavioral disrupter in *A. lacustris* in this important freshwater species cultivated in South America.

## 1. Introduction

The intense exploitation of natural resources resulting from population growth and industrial development generates domestic, agricultural, and industrial effluents that may contain many metals due to the lack of adequate treatment and/or basic sanitation [1,2,3]. Among these metals, it is possible to highlight trace metals such as aluminum, copper, manganese, and zinc [4] (these are metals that, when present in small quantities, may be essential or harmless; however, they can become toxic when their concentrations exceed certain thresholds), as well as toxic metals like lead, mercury, cadmium, and nickel [5], these last ones present significant risks to both animals and humans. Moreover, natural processes, such as weathering and atmospheric deposition, along with anthropogenic activities like mining, smelting, and refining, can significantly alter the concentration of trace metals and toxic metals in aquatic ecosystems, and they can have adverse effects on aquatic organisms [4,6,7]. This factor has a great impact on aquatic environments, which can bioconcentrate in organisms and can be transferred throughout the food chain [8,9], including several teleost fish species, which are important for the food and economy of several countries in the world. Furthermore, several interactions and effects can be caused by anthropogenic chemical compounds, which affect the aquatic ecosystems worldwide, and vary with the amplitude, duration, and persistence of xenobiotics [10].

Among these metals, aluminum (Al) is one of the most abundant in nature. It is the third most common mineral and the most prevalent metallic element in the Earth’s crust, comprising 8.1% of the Earth’s mass (see [4,7,11,12] for review). The natural release of Al into the environment surpasses that from anthropogenic sources. Nevertheless, human activities, such as industrial and municipal discharges, significantly elevate its concentration in surface waters, in addition, aluminum sulfate (Al_2_(SO_4_)_3_) is commonly present in drinking water [7]. Due to its widespread presence and potential toxicity, Al poses a serious environmental threat. It is classified as a hazardous pollutant and can induce toxic effects in both terrestrial and aquatic organisms [12,13]. It is important to note that, in humans, Al has been associated with various neurological disorders, including Alzheimer’s and Parkinson’s diseases, autism, and dementia [7]. In relation to the aquatic environment, the chemistry of Al is different in freshwater and marine systems and can affect animals in different ways as addressed by Botté and colleagues [13]. Moreover, the toxicity in fish varies according to pH, temperature, ions concentration (such as carbon dioxide, oxygen, divalent cations and anions, carbonate alkalinity, salinity), dissolved organic matter, and the time of exposure to the metal [14,15,16]. These water parameters can change the bioavailability of Al, affecting the fish physiology regardless of how it will be organized (such as polymerized, colloidal, or particulate forms) [17,18,19]. Furthermore, Al levels in rivers have risen in recent decades due to factors such as acid rain and industrial pollution. Since the 1980s, it has been recognized that, in conjunction with acidic pH, Al has toxic effects on wildlife, leading to declines in natural fish populations in Scandinavian countries [20]. Due to this increase in Al concentration in the aquatic environment, this metal has been the target of intense scientific research (see [4,7,11,12] for review), but the physiological changes in Neotropical teleost fish species of significance to South American aquaculture require further comprehensive investigation.

Owing to the numerous natural and anthropogenic sources, aluminum is widely present in the environment and can be found in aquatic ecosystems. This metal is associated with various toxic effects, particularly its neurotoxicity in mammalian models. Studies on its impact on aquatic organisms have increased in recent years (see [4,7,11,12] for review). The main effects resulting from exposure to Al in fish are changes in physiological systems, such as the following: (1) osmoregulatory and ionoregulatory processes, caused mainly by cellular changes in the gills and plasma ions, which also compromise the respiratory system [17,21]; (2) metabolic changes, mainly in decreased ovary and plasma proteins, muscle glycogen contents, hepatic lipids due to lipoperoxidation [6,21,22], and consequently, stress oxidative modulation [6,23]; (3) many neuroendocrine dysfunctions lead to changes in reproductive physiology and, therefore, in fish reproduction quality, such as steroid hormones’ (17β-estradiol, testosterone, 11-ketotestosterone, 17α-hydroxyprogesterone, cortisol concentrations) [19,21,24] and thyroid hormones’ (triiodothyronine and thyroxine) [4,22] concentrations; gonadotropin pituitary gene expression [20]; sperm quality, including cytotoxicity and genotoxicity in erythrocytes and spermatozoa [15,25], and consequently spawning and embryo and larval development [26], and also in relative fecundity [19].

The studies above described the effects of Al on fish physiology. Their results can lead to knowledge of the studied species’ reproductive biology, which can lead to complex ecotoxicological evaluations [13]. Nevertheless, assessments of animal social behavior when exposed to Al have still not been addressed so far (mainly in Neotropical teleost fish species), which can also further complete this ecotoxicological knowledge. Aluminum contamination can alter the homeostasis of an organism beyond animal behavior, which is of great importance to be evaluated as it can be used to assess neurotoxicity [27]. It is necessary to investigate the behavioral mechanisms organisms used to adjust to environmental challenges through controlled experiments and studies on the physiological and neurological processes involved [27]. Furthermore, behavior can be related to the species’ survival because the search for food (foraging and competition) [28,29], social interactions, including couple interactions (reproduction behavior, male/female), or even social interactions between the same sex (cooperation and/or agonistic behavior, males/males, and/or females/females), can be altered in a contaminated environment. In this case, research should refine behavioral endpoints in fish and other organisms as sensitive indicators of environmental changes, identifying specific behaviors that respond to stressors like pollutants or temperature shifts [27]. Accompanying this, standardized protocols for measuring these behaviors across species and testing their effectiveness in detecting environmental changes will help understand long-term ecological impacts; and this approach will enhance understanding of behavioral adjustment and its role in environmental monitoring [27]. Moreover, all these expected animal behaviors can generate normal stress response, in addition to the contaminant itself, and consequently change the levels of corticosteroids, such as changes in the plasma cortisol concentration (a stress biomarker) [30,31,32], increasing the serum cortisol concentration response to a stressor is an integral part of the adaptive physiological response, and a disruption in the adaptive physiological response can reduce animal survival [33,34].

Therefore, previous studies showed that Al can be considered as an endocrine disruptor in *Astyanax lacustris*, considering females and males separately [19,24,26]. It can also be considered as a behavioral disruptor in different species, as observed in *Oreochromis niloticus* [7,21], in *Danio rerio* [6], in many salmonid fish [4,35,36], in *Ctenopharyngodon idella*, *Cirrhinus mrigala* [7], and other species (see [7,11] for review). On the other hand, it remains unknown how the social behavior and stress response (using plasma cortisol concentration as a proxy) of *A. lacustris* (females and males together) are affected when exposed to Al. Therefore, this study hypothesized whether Al decreases the social interactions of *A. lacustris* couples after acute exposure (1 h) and whether this metal increases the plasma concentration of cortisol in this important freshwater species cultivated in South America, consumed by the population and requested as “bait” for sport fishing of important commercial species.

## 2. Materials and Methods

### 2.1. Animals and Experimental Design

**Figure 1:**
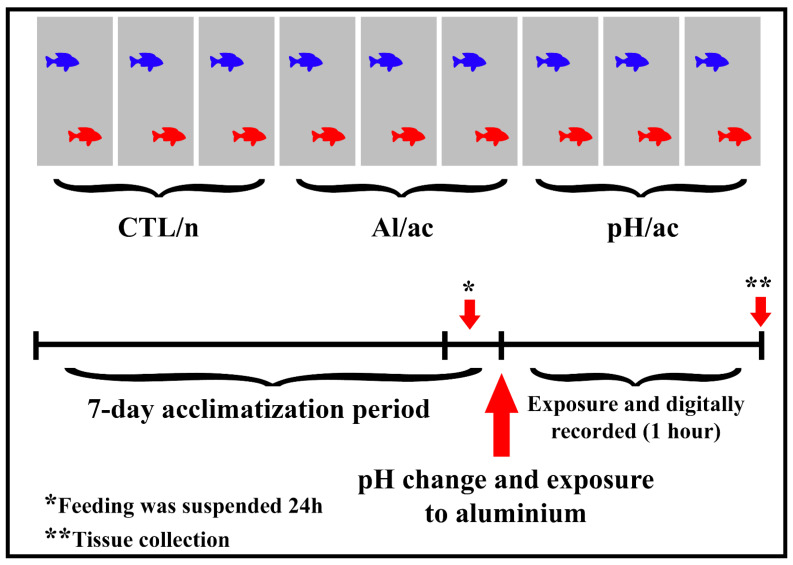
Schematic representation of the experimental design. Exposure of couples (male (blue fish) and female (red fish); *n* = 2) (in triplicate; *n* = 6) of *Astyanax lacustris* in different experimental groups and digitally recorded (1 h): control (CTL/n group) in neutral pH; acid pH (pH/ac group); and aluminum (Al/ac group) in acid pH.

It is worth remembering that a recent taxonomic study Lucena and Soares [37] recognizes the species formerly named *Astyanax altiparanae* as a synonym for *Astyanax lacustris*, the name which is now considered valid for this species. *Astyanax lacustris* is a small omnivorous teleost species (adult average from 10 to 15 cm, possibly reaching up to 60 g) that reproduces mainly during the spring and summer and, usually, transient male sex dimorphism features presenting minor “hooks” during the reproductive season, bringing on roughness to the touch in the anal fin [38,39]. These “hooks” were used as a sexual parameter to separate/identify the sex of the animals. In February and March (reproductive season), sexually mature *A. lacustris* females and males were obtained by the former *Unidade de Hidrobiologia e Aquicultura da Companhia Energética de São Paulo*, CESP, Paraibuna, São Paulo, Brazil (23°24′53.1″S 45°35′59.5″W) and transported to the Ectotherm Bioterium at the Physiology Department, Institute of Biosciences, São Paulo University (Ectotherm Bioterium IB/USP, São Paulo, Brazil). Females and males were maintained in separated black boxes (350 L) for seven days (acclimatization period), with water replenishment (90% every 24 h), and fed daily ad libitum with extruded feed with 32% crude protein, once per day. The water was continuously aerated (always above 6 mg L^−1^), and water temperature (25 ± 2 °C) was monitored daily with a multiparameter probe (ProPlus, YSI, Yellow Springs, OH, USA), which was also used to check the dissolved oxygen. The pH was monitored with a pH meter (Gehaka, São Paulo, SP, Brazil), and a 13:11 light/dark cycle was maintained [38]. Water quality parameters, including nitrogen compounds (NH_3_ and NO_2_^−^), were not quantified in the present study due to the short exposure duration of one hour and the potential for sensor analysis to influence animal behavior. Conversely, in studies involving longer exposure periods (with *A. lacustris*), nitrogen compounds were monitored and analyzed as outlined in a previously published study, which observed no significant variations among the experimental groups [19]. Furthermore, they were deprived of feed for 24 h before beginning the experiments and until the end of the exposure (Figure 1).

The experimental design was carried out using three experimental groups (in triplicate) (Figure 1): control in neutral pH (CTL/n group), acid pH (pH/ac group), and aluminum in acid pH (Al/ac group; 2.0 mg L^−1^). For the pH/ac group, the pH value was acidified with HCl (0.1 N) until 5.5 (mean); and CTL/n in neutral pH was set at 7.0 (mean). For the Al/ac group, the Al solution was obtained using aluminum sulfate (Al_2_(SO_4_)_3_; Sigma-Aldrich Diagnostic INS, St. Lous, MO, USA) from a stock solution prepared with Milli-Q water that was acidified to pH 2.5 with 65% HNO_3_ (Suprapur, Merck, Kenilworth, NJ, USA). As Al is insoluble at pH 6 to 8 [23], an Al group in neutral pH was not considered. Additionally, after adding Al solution in the Al/ac group (nominal concentration of 2.0 mg L^−1^), the pH was adjusted with HCl to 5.5 (average). Therefore, after the acclimation period, the animals (one couple) were transferred to aquariums (60 L each), involved in brown Ethylene-vinyl acetate (E.V.A.), except the front part of the aquariums, because all the interactions were digitally recorded (Everio FullHD JVC, Yokohama, Japan) during 1 h through this region of the aquarium. In this context, fish from different groups and replicas had no visual influence from one pair to the other and not even from people in the Bioterium. Additionally, behavioral records were made between 14:00 and 16:00 h to avoid possible circadian influences.

Furthermore, the concentration of Al used in this experiment is within the range of previous studies. Therefore, the manipulation of aluminum and pH, and the physical-chemical characteristics of this metal in water, have already been established through previous studies in the field [15,19,23,24,25,26]. Moreover, it is known that in the specialized literature, the Environmental Protection Agency of United States (EPA, Washington, DC, USA) recommends a limit of 0.2 mg L^−1^ for Al in water, while in Brazil, the *Conselho Nacional do Meio Ambiente* (CONAMA) has determined that 0.1 mg L^−1^ is the maximum allowed concentration of Al dissolved in water [40]. Despite these recommendations, in the last five years, water quality reports of the *Companhia Ambiental do Estado de São Paulo* (CETESB) indicate that some rivers in the State of São Paulo so far exceed the allowed values, including the Paraíba do Sul River Basin (respectively, min–max 0.10–0.31 mg L^−1^), Córrego do Frutal River, Baixo Tietê River Basin (respectively, min–max 0.05–0.58 mg L^−1^), and Baquirivu-Guaçu River, Alto Tietê River Basin (respectively, min–max 0.10–1.47 mg L^−1^) [41]. Furthermore, some reservoirs show elevated concentrations of dissolved Al that can affect human health, including the Paiva Castro reservoir (0.48–0.63 mg L^−1^ of Al), one of the reservoirs of the Cantareira Complex, which supplies the city of São Paulo, Brazil [42]. Due to these fluctuations of Al in the São Paulo water rivers and the emerging concern of a possible rise in the concentration of this metal as countries develop, the 2 mg L^−1^ concentration is not too far to predict and realistic, as identified in the Ribeirão-Perová, in the Alto Tietê River Basin, where on July 2015 it was 0.49; May 2016, 1.54; July 2017, 12.3; May 2018, 1.88; and November 2019, 3.96 mg L^−1^ [41].

Regarding behavior analysis, the number of agonistic interactions performed by the female and male was recorded. Social encounters and swimming activities were analyzed. As recording times varied slightly between different experimental groups, agonistic interactions are presented as the frequency of a given behavior per ten minutes. Aggressive displays included the time the attacks started, which was recorded. A count of aggressive interactions that consisted of bites, chases (with or without bites), and approaches was also performed. Submissive behavior included escapes (when the fish swims away from the fish that is chasing, biting, or approaching it with a threat) and passive coping (when the fish does not escape or move when bitten or approached). In this study, the term “attack” is used to refer to any of the abovementioned aggressive displays. Moreover, the aquarium was divided into six regions with a permanent marker pen to help observe and quantify the behavior (named, left and right; superior and inferior; front and bottom) to assess the performance of the couple’s swimming activity or the possible escape of an animal or attack. The methodology described for behavioral assessment is grounded in numerous studies previously published in this field [4,6,7,11,43].

All procedures used in the sampling of the animals were in agreement with procedures of the National Council of Experimental experimentation [44] and approved by the Ethics Committee on Animal Use (CEUA) of the Institute of Biosciences, University of São Paulo (Approval code: OF.CEUA/IB/029/2012. REF. 2012.1.762.9 (Protocol number No. 163/2012); approval date: 20 August 2012).

### 2.2. Histological and Hormone Analyses

After 1 h of social interaction digitally recorded, females and males were anesthetized with 0.1% benzocaine (ethyl-p-aminobenzoate), which was previously dissolved in ethanol (10 mL), and placed into a 10 L container with fresh water. Biometrical parameters, total length (cm), and total body mass (g) were registered. Approximately 200–300 uL of blood samples were collected by puncturing the caudal blood vessel using a heparin-coated syringe and centrifuged for 5 min at 655.1 g. The obtained plasma (without hemolysis) was frozen in liquid nitrogen and kept at −80 °C until cortisol concentration analysis. Specimens were euthanized by decapitation at the level of the operculum. Then, the animals were dissected, the liver, ovaries, and testes were removed, weighed to the nearest decigram, and the gonads were fixed in Bouin solution for 24 h and later transferred to ethanol 70% until histological analyses. Both the gonadosomatic index (GSI), which is expressed as the percentage of body mass regarding the gonads [GSI = (gonad weight/total weight) × 100], and the gonads’ histological results were used to confirm the animal sex and to evaluate the gonadal maturation stage according to previous studies [24]. The somatic index of the liver, which is expressed as the percentage of bodyweight regarding the liver [HSI = (liver weight/total weight) × 100], was also calculated.

To conclusively ascertain the sex of the animals and evaluate the reliability of “hooks” as an indicator for sex identification in this study, histological analyses of the gonads was performed at the end of the exposure. For light microscopy, fixed gonad samples were dehydrated through an ascending series of increasing ethanol concentrations and infiltrated with historesin (Historesin Leica). Gonad sections were sliced at 3 μm thick using a microtome (Leica HistoCore AutoCut, Wetzlar, Germany), mounted on Poly-_L_-Lysine solution-coated slides, stained with Schiff periodic acid (PAS)/iron-hematoxylin/metanil yellow [45], and examined and documented using a computerized image analyzer (Leica DM1000 LED light microscope, Leica MC170HD camera, and computer image capture Leica LAS Interactive Measurements, Wetzlar, Germany).

As plasma cortisol concentration may quickly increase due to fish manipulation, we quantified cortisol concentration from blood samples whose drawing time was less than 4 min after netting at the aquarium [46]. Thus, the plasma levels of cortisol were quantified by an enzyme-linked immunosorbent assay (ELISA) (IBL International, Hamburg, Germany). Pilot assays were performed using three different dilutions of three samples to establish the appropriate working dilutions for cortisol concentration for this species (including females and males). A standard curve was conducted for each ELISA plate. Working dilution was 1:4 for both females and males. In all cases, samples were assayed in duplicate, and analyses were carried out on samples whose coefficients of variation were ≤20% [47], following the manufacturer’s instructions. Intra-assay variation (minimum and maximum) was 1.0 and 18.7%, while inter-assay variation (minimum and maximum) was 5.1 and 10.85%. The detection limit of the assay was 2.46 ng/mL. According to previous studies, ELISA hormone assays were validated for *A. lacustris* [19,24].

### 2.3. Statistical Analysis

All values were expressed as the mean ± standard error of the mean (M ± SEM) and subjected to the Kolmogorov–Smirnov test to verify the normality and homogeneity variance (Levene’s test) were previously verified. Grubb’s test (GraphPad) was used to verify outliers. The underlying assumptions of homogeneity and normality were verified prior to analysis, and data with a Gaussian (normal) distribution were analyzed using parametric tests. Conversely, when the data did not meet the assumption of homogeneous variances, nonparametric methods were employed for comparison. Statistical analysis of behavioral parameters, plasma cortisol concentration, and organ-somatic indexes was compared by the one-way analysis of variance (ANOVA) followed by the Tukey’s test (parametric comparisons). If data were nonparametric, a Kruskal–Wallis test, followed by Dunn’s test, was performed. In all cases, a significance level of *p* < 0.05 was considered statistically significant (considering a couple in the same group (female vs. male) or the same sex in different experimental groups (male vs. male; female vs. female)). All statistical analyses were performed using the SigmaStat software for Windows (version 3.10).

## 3. Results

### 3.1. Water Analysis

The water quality parameters (temperature, dissolved oxygen, and pH) during experimental design are shown in Table 1.

### 3.2. Animals and Biometrical Parameters

**Figure 2:**
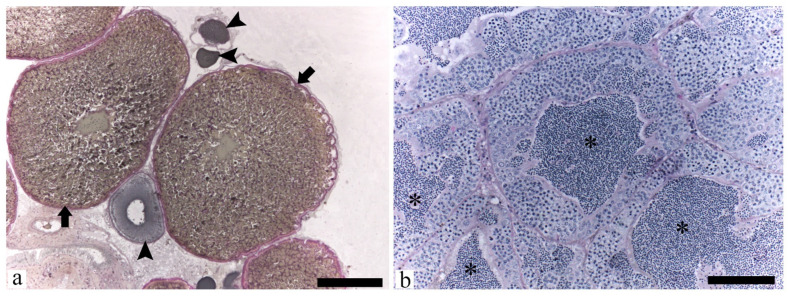
Histological sections of *Astyanax lacustris* gonads. (**a**) mature female showing vitellogenic oocytes (arrow) and perinucleolar oocytes (arrowhead); (**b**) mature male showing several ducts full of sperm (asterisk). Stain: Schiff periodic acid (PAS)/iron-hematoxylin/metanil yellow. Bar: (**a**) 200 μm; (**b**) 100 μm.

Macroscopically, all males presented minor ‘hooks”, bringing roughness to the touch in the anal fin, and histology methods confirmed the sex (100% accuracy of sex with hooks). Histologically, females presented vitellogenic oocytes and some perinucleolar oocytes (Figure 2a), and males showed several ducts full of sperm (Figure 2b). In this sense, all animals were adults and sexually mature. There were no qualitative morphological differences between ovarian and testicular development among animals from different experimental groups.

During the exposure period, the survival rate in all groups was 100%. As expected, all females (in all groups) were larger and had a higher body mass than males (Table 1) (respectively, CTL/n: *p* = 0.004 and *p* = 0.004; Al/ac: *p* = 0.003 and *p* = 0.004; pH/ac: *p* = 0.002 and *p* ≤ 0.001). Furthermore, GSI was also higher in females than in males (Table 1) (within the same experimental group; CTL/n: *p* = 0.004; Al/ac: *p* = 0.033; pH/ac: *p* = 0.043). Nonetheless, there were no differences in biometric data among females or males in the different experimental groups (Table 1), even in the GSI and HSI indexes (Table 1).

### 3.3. Social Behavior

**Figure 3:**
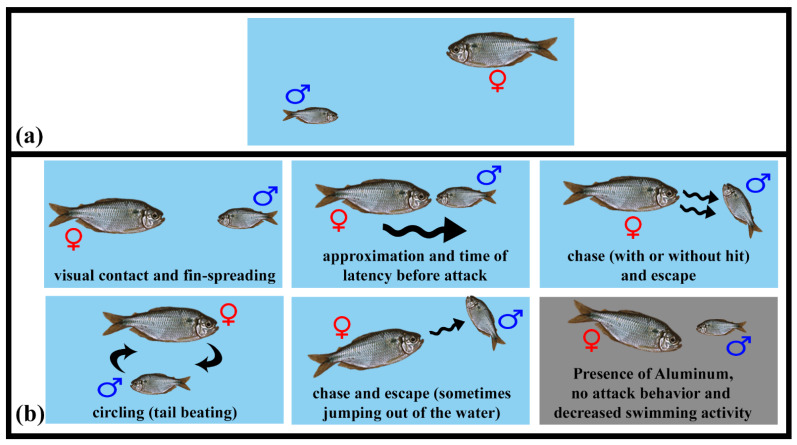
Evaluating the aggressiveness in *Astyanax lacustris*: (**a**) behavioral analysis of a couple in the experimental aquarium (60 L); (**b**) behaviors observed in *A. lacustris* (a couple, with a more aggressive female and bigger than the male) in experimental aquariums; the last gray box represents the aquarium with aluminum and “cloudy water”, and its main behaviors. For more information, see the text.

**Figure 4:**
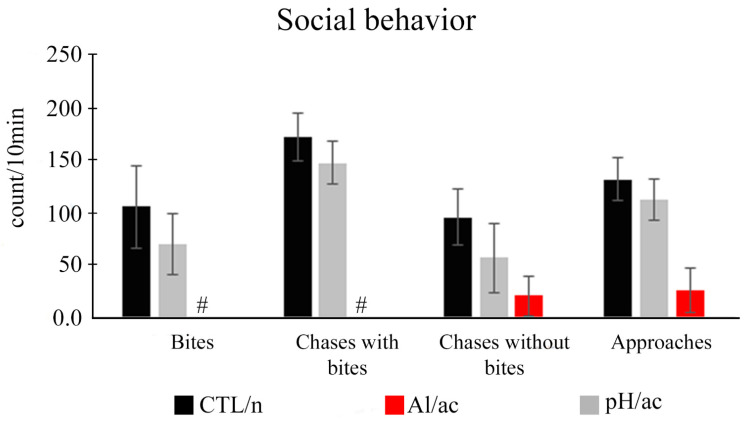
General social behavior of *Astyanax lacustris* females and males in different experimental groups: control (CTL/n) in neutral pH; aluminum in acid pH (Al/ac); acid pH (pH/ac). Number (based on 10 min) of bites, chases with bites, chases without bites, and approaches. Data are presented as the mean ± standard error of the mean (M ± SEM). # indicates no behavior in this group.

**Figure 5:**
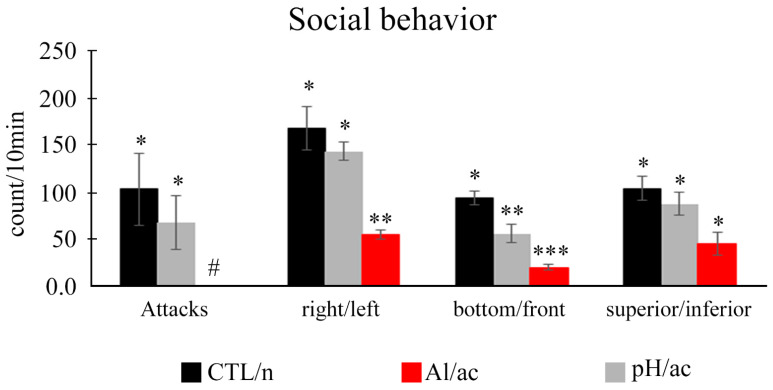
Social behavior of *Astyanax lacustris* females. Number (based on 10 min) of attacks and movements (right/left; bottom/front; superior/inferior) in different experimental groups: control (CTL/n) in neutral pH; aluminum in acid pH (Al/ac); acid pH (pH/ac). *, **, *** Asterisk indicates a statistical difference between experimental groups (right/left: CTL/n (*p* < 0.006) and pH/ac (*p* < 0.004) groups compared with Al/ac group; bottom/front: CTL/n compared with pH/ac (*p* < 0.022); CTL/n compared with Al/ac (*p* < 0.001); pH/ac compared with Al/ac (*p* < 0.025)); # indicates no behavior in this group. Data that were not statistically significant *p* > 0.05.

As females were larger than males, this characteristic was considered to differentiate animals during behavioral analyses. In general, one ethogram was elaborated on the social behaviors (social interactions) presented by *A. lacustris* in females and males in different experimental groups. It was presented in Table 2 and Figure 3, Figure 4 and Figure 5. Once fish were placed in the experimental aquarium, a couple showed gregarious behavior over the bottom (always) right or left corner of the aquarium (all experimental groups). Over time, the females in the CTL/n and pH/ac groups were more active (with more aggressive interactions) than males, unlike the Al/ac group, in which the couples were less active and had more opercular beats (data not quantitatively quantified, only qualitatively). *A. lacustris* females were more aggressive, including attacks with bites, than males in the CTL/n and pH/ac groups. In the Al/ac group, there were no attacks from females to males, only chases without bites and some approaches without threats. Briefly, the females in the CTL/n and pH/ac groups showed the following behaviors into males: bites (respectively, 103.7 ± 38.4 and 68.5 ± 28.6); chases with bites (respectively, 168.7 ± 21.9 and 144.3 ± 20.1); chases without bites (respectively, 94.0 ± 26.5 and 56.0 ± 31.9); and approaches (respectively, 129.0 ± 19.9 and 110.0 ± 18.9). Additionally, the majority of bites were generally concentrated on the lateral regions of the body and the caudal fin (86.8%), with occasional occurrences on the eyes or cranial region (13.2%). On the other hand, the females in the Al/ac only presented chases without bites (20.3 ± 19.2); and approaches (26.0 ± 21.0) (Figure 4). No bites or chases with bites were observed in this group.

When the fish were placed in the experimental groups, the time elapsed for the first female attack on the males was 11.6 ± 4.92 s for the CTL/n group and 10.5 ± 2.17 s for the pH/ac group. No first attack was observed in the Al/ac group. Submission displays were observed in the males in the different experimental groups (Table 2 and Figure 3). In the CTL/n and pH/ac groups, males were active, but, in the escape direction (male swims away from female). On the other hand, in the Al/ac group, males displayed passive coping, they did not escape or move, remaining almost stationary in the same place (Figure 4). In all experimental groups, *A. lacustris* males did not attack, threaten or chase the females. It is worth noting that in the Al/ac group, two different factors were observed (Table 2 and Figure 3): (1) movement to the surface of the aquarium (sudden rise in the “water surface”); (2) high mucus production, which made the aquarium water “cloudy/opaque”.

In this sense, *A. lacustris* females were more aggressive and, consequently, more active than males. Therefore, they dictated the rhythm of activity in the experimental aquarium. The swimming activity analysis focused on them. Regarding the female attacks on the males, in just 10 min at the beginning of the recording, a total of 103.66 ± 38.44 direct attacks were counted in the CTL/n group and 70.00 ± 28.52 direct attacks in the pH/ac group (*p* = 0.498). No attack was observed during 1 h of recording in the Al/ac group (Figure 5). Considering the couple’s movement in the aquarium, CTL/n and pH/ac groups generally moved more than the Al/ac group. Therefore, moving from right/left or left/right was greater in the CTL/n (*p* < 0.006) and pH/ac (*p* < 0.004) groups than in the Al/ac group, and no difference was identified between CTL/n and pH/ac (*p* = 0.073) (Figure 5). For the front/bottom or bottom/front movement, differences were observed between the three experimental groups (CTL/n and pH/ac, *p* < 0.022; CTL/n and Al/ac, *p* < 0.001; pH/ac and Al/ac, *p* < 0.025) (Figure 5). There were no significant differences between groups for superior/inferior or inferior/superior (*p* = 0.812) (Figure 5). Therefore, we can conclude that animals were more active in the CTL/n and pH/ac than in the Al/ac experimental group.

### 3.4. Hormone Analyses

**Figure 6:**
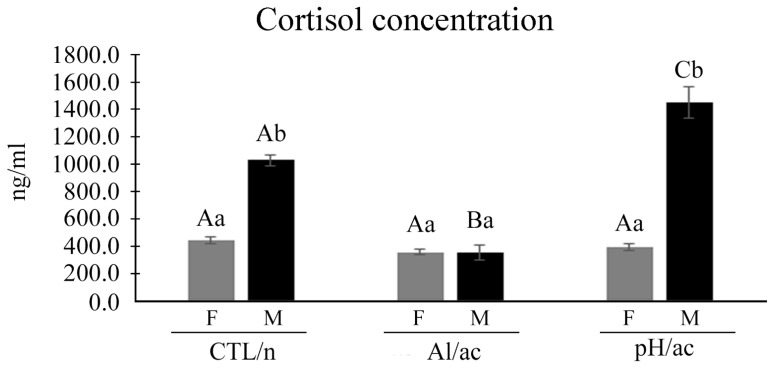
Plasma cortisol concentration in *Astyanax lacustris* female (F) and male (M) in different groups: CTL/n (control group in neutral pH); Al/ac (aluminum group in acid pH); pH/ac (acid pH group). Data are presented as the mean ± standard error of the mean (M ± SEM). ^A,B,C,a,b^ Different uppercase letters indicate differences between females and males in different groups (males in CTL/n (*p* < 0.011) and pH/ac (*p* < 0.001) groups compared with Al/ac group; pH/ac (*p* < 0.038) compared with CTL/n group), and lowercase letters indicate differences between females and males in the same group (CTL/n (*p* < 0.001) and pH/ac (*p* = 0.004) groups compared with Al/ac group). Data that were not statistically significant *p* > 0.05.

In general, the cortisol plasma concentration in *A. lacustris* females and males from the same group and in different experimental groups was distinct (Figure 6). Comparing a couple, in the CTL/n and pH/ac groups, males had a higher plasma cortisol concentration than females (respectively, *p* < 0.001 and *p* = 0.004). On the other hand, no statistical difference was observed between males and females in the Al/ac group (*p* = 0.907). Furthermore, considering the different groups, females did not show significant statistical differences (*p* = 0.063). On the other hand, males in CTL/n and pH/ac groups showed higher concentrations of plasma cortisol than in the Al/ac group (respectively, *p* < 0.011 and *p* < 0.001). Moreover, a higher plasma level was observed in males from the pH/ac group than in the CTL/n group (*p* < 0.038).

## 4. Discussion

The social behavior of *A. lacustris* couples in captivity was described for the first time, and it was observed that females are more aggressive than males. This type of agonistic behavior presented by females is also observed in other teleost species, such as *Danio rerio* [48], *Cichlasoma nigrofasciatum*, *Eucyclogobius newberryi*, and *Sygnathus typhle* [49]. Unlike in *Cichlasoma dimerus* [50], *Astotilapia burtoni* [51], and *Betta splendens* [52], males are more aggressive than females. It is noteworthy that *B. splendens* males have undergone an extensive history of artificial selection, specifically targeting exceptionally high levels of intraspecific aggressive behavior [53]. Moreover, this study revealed that the presence of Al can decrease the movement of animals, including the influence of a couple’s exposure to Al and its consequences on this social behavior and plasma changes in the cortisol concentration, one of the main stress indicators in fish. Females cause so much stress (by aggressive social behavior) in males, which could be the reason of higher plasma cortisol concentration in males than females. It was shown that Al altered the aggressive behavior of females, which could trigger a decrease in plasma cortisol concentration in *A. lacustris* males to the point that couples have the same cortisol concentration profile. Two different factors in Al exposed animals attracted attention in this study: (1) the high production of mucus, which made the water cloudy; and (2) the apparent increase in the opercular beat and sudden ascents to the surface of the aquarium. Therefore, it is suggested that this high mucus production is an attempt to avoid further Al contamination since there may be relevant immune molecules in the mucus [54]. Furthermore, as reviewed by Reverter and collaborators [55] mucus serves as a dynamic physical and biochemical barrier, fulfilling a wide range of biological and ecological functions. These include osmoregulation, defense against mechanical abrasion, shielding from environmental toxins and metal exposure, facilitation of parental feeding, protection against pathogenic organisms, and enabling chemical communication. Additionally, the increase in the opercular beat and the fish’s attempt to breathe oxygen on the surface suggest that Al can influence respiratory physiology. This hypothesis corroborates the results obtained by Allin and Wilson [56], showing that Al interferes in the respiratory system, restricting the respiratory scope.

Behavior represents the manifestation of an organism’s motivational, biochemical, physiological, and environmental state at the organismal level. It has been demonstrated that sublethal metal toxicity can lead to alterations in various aspects of fish behavior [57]. The behaviors assessed following metal exposure include avoidance/attraction, activity, critical swimming performance, respiratory behavior, learning, intraspecific social interactions, reproductive behavior, feeding behavior, and predator avoidance [58]. Laboratory tests conducted with high-quality dilution water and unexposed fish are likely to overestimate the fish’s responsiveness to metal contamination in natural environments [59]. Growth, along with survival and reproduction, remains one of the most crucial endpoints used to assess the impact of toxicants on fish [58]. Taking into account the reproductive aspects, in a previous study, it was observed that *A. lacustris* males exposed to Al for 24 h, and plasma cortisol concentration did not change, despite a downward trend in these animals [24]. On the other hand, Al altered the plasma profile of other gonadal steroids, such as 11-ketotestosterone concentration. Nevertheless, *A. lacustris* females exposed for 96 h to Al showed a decrease in cortisol and 17α-hydroxyprogesterone plasma concentrations [19]. However, both studies evaluated each gender of *A. lacustris* separately. According to these results, the subsequent question was “how did *A. lacustris* couples behave together in response to exposure to metal Al?” in response to stress (using plasma cortisol concentration analysis as a proxy) and behavioral responses. In this case, the couple presented a decreased cortisol plasma concentration due to the presence of Al. Nevertheless, in *A. lacustris* males, this decreased cortisol plasma concentration can also indicate the females’ lack of aggressive behavior. Moreover, *O. niloticus* females exposed to Al for 96 h also showed a decrease in 17α-hydroxyprogesterone and cortisol plasma concentrations [21]. The reviews of Alasfar and Isaifan [12] and Botté and collaborators [13] describe other aquatic species in which Al was studied and considered a disruptor. Therefore, all results obtained by these studies suggest that Al is an endocrine disruptor, and the results obtained in this study add that it could be also a behavior disruptor.

Furthermore, *A. lacustris* couples’ exposure to Al/ac showed gregarious behavior, no aggressive interactions (such as attacks), little swimming movement, and sudden ascent to the surface of the aquarium. On the other hand, control and acid pH groups presented several agonistic and aggressive behaviors, without a difference between the number of overt and restrained aggressions. Using other endocrine disruptor as pesticides, Cuña and collaborators [60] showed that when *C. dimerus* individuals were exposed to organochlorines (endosulfan) (96 h), they exhibited suppression of spontaneous locomotor activity, hyper-excitability to external stimuli, darkening of the skin, spasms, and scoliosis. Moreover, the fish would remain on their side on the bottom of the aquaria and occasionally swim rapidly and erratically around it, colliding with the tank walls, suggesting this pesticide’s neurotoxicity due to these behavioral changes. None of these types of behavior were observed in *A. lacustris* animals in this study, except for the suppression of spontaneous locomotor activity. On the other hand, the possibility of Al neurotoxicity cannot be ruled out, as other studies indicate that Al is a cytotoxic and genotoxic compound in *A. lacustris* [25]. Furthermore, neurotoxic studies, such as the activity of the enzyme acetylcholinesterase (AChE), hematological parameters, and others, need to be performed to confirm this hypothesis. Still, in the movement of animals, metals such as Al can affect the locomotor activity in many ways: (a) alter sensory perception and reduce responses to normal olfactory cues associated with activities such as feeding, mate selection, or homing (all having a component of locomotor behavior); (b) cause alterations in free locomotor activity, manifested as hypoactivity or hyperactivity; (c) alter locomotor components such as turning frequency or angular orientation; and (d) reduce swimming performance [43]. The most studied locomotor response to metal contamination is the alteration in free locomotor activity, manifested as hyperactivity or hypoactivity (this last one, as presented here), and a specific test of “elicit movement” toward (attractance) or away from (avoidance) a contaminated area, simply by changing the swimming behavior. It is also important to note that the quantification of Al in different tissues of *A. lacustris* was not an objective of the present study, as longer exposures to Al in this species have already been assessed and published in previous studies, including gills, brain, liver, gallbladder, white muscle, kidneys, spleen, and ovaries [19].

It is important to mention that when females arrived and were placed in the black boxes, they were more active and aggressive among themselves during the acclimatization period, differently from males, which were calmer and less active. These behavior patterns among females (aggressive) and males (calmer) of *A. lacustris* described herein were observed in previous studies, confirming that it is easier to handle males than females [22,23,24,61]. Furthermore, this aggressiveness pattern of females towards males of *A. lacustris* was definitive in changing this experimental design’s conditions because in the pilot trials, when a couple of this species was placed in an aquarium with a volume of 30 L, females killed the males in less than 1 h. Moreover, females chase many males, often swimming rapidly, frequently colliding with the aquarium walls, and end up being seriously injured, to the point of dying. Females probably kill males due to space restrictions for males to escape. Thus, the water volume in the aquaria had to be increased to 60 L to carry out this study. Therefore, due to previous experience (since 2010 [58] in studying *A. lacustris*, and always observing that females were/are more aggressive than males and added to pilot tests (nine times with couples in 30 L), this behavioral pattern of *A. lacustris* was considered, and the animal number was decreased according to the 3Rs principle (reduction, substitution and refinement), as suggested by Tannenbaum and Benett [62] (theory of Russell and Burch’s 3Rs [63]).

Several studies indicate that females of *A. lacustris* are larger than males [38,39], and this characteristic was valid as a sexual dimorphism to assess the social behavior of the couple. According to Maruska and Fernald [51] and Magurran and Garcia [49], size is the most important factor in establishing social hierarchy, probably related to strength, which is supported by the observation that aggressive interactions, including physical contact, establish hierarchies. Similarly, corroborating this biometrical data, females showed higher GSI values than males (this study), such as several other studies comparing females and males in other teleost species [38,64,65,66]. Nevertheless, as anticipated, no significant differences were observed between the experimental groups, likely due to the brief duration of exposure to Al; differently from that identified in long exposures of 96 h to metals, as observed in *Notopterus notopterus*, in which GSI values decreased [61]. On the other hand, several fish species showed coloration patterns associated with social and reproductive behavior, as noted in *C. dimerus* [50], *A. burtoni* [51], and others teleost species [67], which differs from *A. lacustris*, which does not show color pattern variations in any period of the life cycle. Therefore, these minor “hooks”, total length, and total body mass can be used as morphological characteristics to distinguish males from females of *A. lacustris*, confirmed by gonadal histology.

## 5. Conclusions

In the present study, results suggest that Al (2.0 mg L^−1^) can trigger physiological alterations in *A. lacustris* couples, even in a short period, such as acute exposure of 1 h (acute stress response). Although new studies investigating other physiological parameters integrated into behavior are necessary, especially in aquatic systems, the changes evaluated in this study may negatively affect the animal homeostasis of this species, notably animal stress (cortisol pathway), and social behavior, which are important for survival, reproduction physiology, and environmental ecology. Thus, the hypothesis is confirmed that Al can also be considered as a behavioral disruptor in *A. lacustris* females and males. On the other hand, further studies investigating other physiological parameters integrated with behavior are required, especially in aquatic systems, presenting an opportunity for future research.

## Figures and Tables

**Table 1:** Biometrical parameters, total length (TL) and total body mass (TW); gonadosomatic (GSI) and hepatosomatic (HSI) indexes of *Astyanax lacustris* female and male, and water quality parameters, dissolved oxygen, pH, temperature in different groups: CTL/n (control group in neutral pH), Al/ac (aluminum group in acid pH), pH/ac (acid pH group) at the beginning of exposure.

		CTL/n		Al/ac		pH/ac	
		Female	Male	Female	Male	Female	Male
Biometric Parameters	TL (cm)	12.16 ± 0.44 ^Aa^	10.50 ± 0.50 ^Ab^	12.17 ± 0.17 ^Aa^	10.00 ± 0.29 ^Ab^	12.83 ± 0.17 ^Aa^	10.50 ± 0.29 ^Ab^
	TW (g)	27.35 ± 2.95 ^Aa^	15.36 ± 3.16 ^Ab^	25.13 ± 2.01 ^Aa^	11.48 ± 0.97 ^Ab^	29.15 ± 0.90 ^Aa^	13.61 ± 1.15 ^Ab^
	GSI (%)	8.70 ± 0.71 ^Aa^	2.61 ± 0.16 ^Ab^	11.72 ± 2.87 ^Aa^	2.46 ± 0.32 ^Ab^	13.30 ± 2.48 ^Aa^	2.21 ± 0.88 ^Ab^
	HSI (%)	0.78 ± 0.51 ^A^	0.71 ± 0.44 ^A^	0.81 ± 0.17 ^A^	0.66 ± 0.92 ^A^	0.69 ± 0.78 ^A^	0.67 ± 0.78 ^A^
Dissolved oxygen (mg/L^−1^)		6.18 ± 0.20		6.35 ± 0.40		6.16 ± 0.20	
pH		7.27 ± 0.01		5.33 ± 0.10		5.37 ± 0.01	
Temperature (°C)		24.30 ± 0.20		24.97 ± 0.10		24.93 ± 0.50	

Values (mean ± standard error of the mean). ^a,b^ Different letters are statistically different between female and male in the same group (total length: CTL/n: *p* = 0.004; Al/ac: *p* = 0.003, and pH/ac: *p* = 0.002; total body mass: CTL/n: *p* = 0.004; Al/ac: *p* = 0.004, and pH/ac: *p* ≤ 0.001; gonadosomatic index: CTL/n: *p* = 0.004; Al/ac: *p* = 0.033, and pH/ac: *p* = 0.043). ^A^ Different letters are statistically different between female and male in different group. Data that were not statistically significant *p* > 0.05.

**Table 2:** Ethogram elaborated from the social behaviors (social interactions) presented by *Astyanax lacustris* female and male, and consequences on water quality in different experimental groups.

*Astyanax lacustris* female		
Social interactions	Types	Behavior
Aggressiveness display	Physical contact	Bites with the mouth ^(1,2)^
		Tail hit ^(1,2)^
		Chases with attack ^(1,2)^
	Threats	Chases without attack ^(1,2)^
		Approaches ^(1,2)^
Passive coping	No threat	No sudden moves ^(3)^
*Astyanax lacustris* male		
Social interactions	Types	Behavior
Submission display	Activity	Escape (fish swim away) ^(1,2)^
	Passive coping	Did not escape or moved ^(3)^
*Astyanax lacustris* couple		
Social interactions	Types	Behavior
Swimming activity	Active/Inactive	Swimming: aquarium (left/right) ^(1,2)^
		Swimming: aquarium (superior/inferior) ^(1,2)^
		Swimming: aquarium (front/bottom) ^(1,2)^
		Sudden rise in the water surface ^(3)^
Water quality	Clear/Opaque	Clear ^(1,2)^
		Opaque (cloudy): mucus production ^(3)^

Consequences on water quality exhibited by control group in neutral pH ^(1)^; acid pH group ^(2)^; and aluminum group in acid pH ^(3)^.

## Data Availability

Data available from the authors by request.
